# Predictors of triage pain assessment and subsequent pain management among pediatric patients presenting to the emergency department

**DOI:** 10.1371/journal.pone.0296240

**Published:** 2023-12-21

**Authors:** Rahim Valani, Fazila Kassam, Shauna Jose, Mario Hanna, Tanmay Sharma, Jhanahan Sriranjan, Yazad Bhathena, Umairah Boodoo, Aashna Agarwal, Suneel Upadhye

**Affiliations:** 1 University of Toronto, Toronto, Canada; 2 University of British Columbia, Kelowna, Canada; 3 McMaster University, Hamilton, Canada; 4 Western University, London, Canada; Medical University of Vienna, AUSTRIA

## Abstract

**Background:**

Pediatric patients with pain of various causes present to the emergency department. Appropriate assessment and management of pain are important aspects of emergency department treatment. However, only a few studies have identified the predictors of both outcomes. This study aimed to evaluate the rate of pain assessment at triage and subsequent management and to identify the predictors of each outcome.

**Methods:**

This was a multi-center retrospective study based at five community emergency departments. Pediatric patients (< 18 years) with pain or injury who presented to the emergency department between February 2018 and May 2018 were included. In addition to patient demographics, the initial pain assessment at triage, reason for visit, and time to analgesia were determined. Further, the type and route of analgesia were identified in patients who received analgesia. Univariate and multivariable regression models were used to identify predictors of pain assessment and management.

**Results:**

There were 4,128 patients with an average age of 9.6 years, and 49.1% of them were female. Only 74.2% of the patients underwent assessment for pain at triage, and 18.3% received analgesia. The median time to analgesia was 95 (IQR: 49–154) min. Most patients presented with head/neck (36.1%), upper limb (21.6%), and lower limb (19.9%) pain. The oral route was the most common analgesia delivery method (67.4%), and ibuprofen and acetaminophen were the primary agents used. Younger age, higher acuity, and presenting with head or neck pain were independent predictors of pain assessment at triage, while children 3–5 years and those with lower extremity pain were more likely to receive analgesia.

**Conclusion:**

Although pain assessment at triage has improved in pediatric patients, there is still a major deficiency in adequate pain management. Our study highlights predictors of pain assessment and management that can be considered for improved pediatric care.

## Introduction

Pain is a common complaint among pediatric patients presenting to the emergency department (ED). In 2017, 19.7 million pediatric visits to EDs in the US were related to pain [1]. Despite ongoing advocacy for pain assessment and management of children in the ED, there is still a major limitation in achieving these goals [2–5].

Effective pain management requires an initial assessment that is age-specific, an intervention that will help reduce pain, and re-evaluation that can identify pain improvement or the need for further treatment. Pain is a complex process that makes it challenging to evaluate, particularly in nonverbal patients. The three dimensions for measuring pain are as follows: self-reporting, behavioral, and physiological responses [4, 6]. Self-reporting is the most reliable method in children who have good cognitive function and can quantify pain [4, 7, 8]. Infants and young children express pain through observed behaviors, which can be a challenge for general ED triage nurses who may not be comfortable with such assessments. Finally, there are physiological parameters which reflect the stress response from pain resulting in changes in some of the vital signs, including heart and respiratory rates [9].

Inadequate pain management in pediatric patients may result in long-term negative effects, including distress during medical procedures, pain tolerance, and altered pain response [6, 10]. ED triage assessment is critical for pain assessment and initial management [11]. It is the first point of contact with the patient, and it provides the best opportunity to start analgesia while the patient is waiting to be assessed by a physician. Barriers to pain management include difficulties in pain assessment of preverbal children, diagnostic uncertainty, fear of masking important clinical signs, concern about medication effects, lack of time, poor knowledge of medical staff, and inadequate space for assessment or monitoring [3, 12, 13].

Many countries and international organizations have established policies regarding the issue of pediatric pain, and in particular Italy has disseminated specific laws to ensure that pain relief is a child’s right to health [14–17]. Pediatric patients presenting to the ED in Italy must undergo pain assessment completed at first contact and be treated promptly. Previous studies have shown that the rate of pain assessment or treatment is poor in pediatric patients presenting to the ED in small cohort, and only a few studies have identified the predictors of both outcomes [3, 5, 9, 12, 18–20]. Furthermore, these studies are based on specialized pediatric centers, which do not make them generalizable to community EDs. This study aimed to evaluate the rates of pain assessment and management at triage and to identify the predictors of each outcome in the community.

## Materials and methods

This retrospective chart review was conducted at five community health care facilities in a semirural area in southeastern Ontario, Canada. Pediatric patients aged <18years who presented to any of the five sites under the Niagara Health System (NHS) between February 1, 2018 and May 31, 2018 were included in the analysis. Patients were identified through the Canadian Emergency Department Information System based on the presenting complaint list, and those presenting with either “pain” or “injury” were selected. The NHS has a catchment population of approximately half a million residents, and it comprises the Greater Niagara General Hospital, St. Catherine’s Hospital, Welland Hospital, Douglas Memorial (Fort Erie) site, and Port Colborne site. This study was approved by the Hamilton Integrated Research Ethics Board (7428-C), and the need for informed consent was waived due to the retrospective nature of the study.

Data on patient’s age, sex, triage acuity (based on the Canadian Triage and Acuity Scale [CTAS]), and pain assessment on the child performed by a triage nurse were reviewed from the medical charts. The physician’s initial assessment times were defined as the registration time to the time when the patient was seen by a physician. In patients who received analgesia, the type of medication, route of administration (oral, topical, intravenous, intramuscular, or intranasal), and the time when the first dose was administered were recorded.

Several authors independently reviewed the charts, and 10% were reviewed by a senior abstractor (FK) to ensure the integrity of data collection. The inter-rater reliability ƙ was 0.85. For any discrepancies, a senior emergency physician (RV) reviewed the chart, and the team reached an agreement based on consensus.

Our primary outcomes were to identify predictors of pain assessment and subsequent treatment in pediatric patients presenting to the ED. Predictors were selected based on information easily identified at triage.

All statistical analyses were performed using the Statistical Package for the Social Sciences version 25 (IBM, Armonk, NY). Descriptive analysis was performed to assess the distribution of traits within the study sample. Data for continuous variables were expressed as mean and standard deviation, median and interquartile range, and maximum and minimum values. Meanwhile, categorical data were evaluated using counts/frequencies and percentages. Age was stratified into three categories: ≤ 2 years, 3–5 years, and ≥ 6 years. Univariate regression analysis was conducted to evaluate the relationship between selected factors (sex, age group, CTAS score, and location of pain) and two outcomes (assessment of pain and treatment of pain). These variables were also subsequently assessed in a multivariable regression model to evaluate the adjusted effect of each factor in relation to the outcome variables. Age ≥ 6 years and abdominal pain were selected as the reference categories for the multivariate analysis. A P-value of < 0.05 was considered statistically significant.

## Results

During the study period, there were 196,948 patient visits across the five sites, and 15.7% were pediatric patients. Of these, 4,128 pediatric patients came to the ED with a presenting complaint of pain. The patients’ average age was 9.61 (standard deviation: 5.15) years, and 49.1% (n = 2,025) of patients were female. Most patients were classified as CTAS level 3 (57.5%). The median time to assessment by a physician was 65 (IQR: 31–118) min (Table 1).

**Table 1:** Demographic characteristics of the patients.

	Total (n = 4128)	Assessed (n = 3063)	Managed (n = 764)
	Frequency (%)	Frequency (%)	Frequency (%)
Sex			
Male	2101 (50.9)	1528 (49.9)	388 (50.8)
Female	2025 (49.1)	1533 (50.1)	376 (49.2)
CTAS			
1	38 (0.9)	28 (0.9)	18 (2.4)
2	995 (24.1)	866 (28.3)	303 (39.7)
3	2372 (57.5)	1808 (59.0)	363 (47.5)
4	515 (12.5)	302 (9.9)	60 (7.9)
5	208 (5.0)	59 (1.9)	20 (2.6)
Age			
≥ 6 years	3005 (72.8%)	2476 (80.8)	543 (71.1)
3–5 years	655 (15.9%)	407 (13.3)	152 (19.9)
≤ 2 years	468 (11.3%)	180 (5.9)	69 (9.0)
Location of Pain			
Abdomen	629 (15.2)	508 (16.6)	145 (19.0)
Back	74 (1.8)	64 (2.1)	17 (2.2)
Chest	138 (3.3)	118 (3.9)	23 (3.0)
GU/Pelvic	63 (1.5)	44 (1.4)	13 (1.7)
Head and Neck	1490 (36.1)	921 (30.1)	292 (38.2)
Lower Extremity	823 (19.9)	689 (22.5)	116 (15.2)
Other	18 (0.4)	11 (0.4)	4 (0.5)
Upper Extremity	891 (21.6)	707 (23.1)	154 (20.2)

*two missing for sex, two missing for location of pain; CTAS = Canadian Triage and Acuity Scale; GU = Genitourinary

From the cohort, 74.2% of patients were assessed for pain, and only 18.5% received analgesia. The most common pain locations were the head/neck (36.1%), upper extremities (21.6%), and lower extremities (19.9%), and these locations were also most assessed. Of the patients assessed, those having pain in the head / neck and genitourinary/pelvic areas had a higher rate of receiving analgesia. The primary pain treatment modality was oral analgesics, with acetaminophen and ibuprofen being the two most common agents used in 62.0% of patients (Table 2).

**Table 2:** Type of analgesia used for pain management among pediatric patients.

Analgesic use	Percentage (%) of patients who received analgesia
Acetaminophen	215 (23.5%)
Ibuprofen	352 (38.5%)
Ketorolac	105 (11.5%)
Morphine	56 (6.1%)
Hydromorphone	2 (0.2%)
Fentanyl	4 (0.4%)
Others	180 (19.7%)
**Total**	914*

*Some patients might have received more than one analgesic agent.

Univariate analysis showed male patients, children ≥ 6 years, high acuity (lower CTAS), and those with pain in the abdomen, chest, or extremities were more likely to be assessed (See Table 3). Meanwhile, those between ages 2–5 years, higher acuity, and abdominal pain were more likely to be treated. Table 4 shows the multivariable logistic regression for pain assessment and management. Age ≥6 and abdominal pain were used as the reference category for age and location of pain respectively. Patients with age ≤ 2 years (OR 0.148; 95% CI: 0.117–0.188), age between 3–5 years (OR 0.410; 95% CI: 0.333–0.505), high acuity patients (OR 0.391; 95% CI: 0.35–0.44), head / neck pain (OR 0.615; 95% CI: 0.474–0.798) were less likely to be assessed for pain, while those with lower extremity pain (OR 1.547; 95% CI: 1.13–2.111) were more likely. Pain treatment was significant only for age 3–5 years (OR 1.413; 95% CI: 1.139–1.752) and acuity based on CTAS (OR 0.528; 95% CI: 0.470–0.594).

**Table 3:** Predictors of assessment and management.

Predictor	Pain Assessed		Pain Treated	
	OR (95% CI)	P value	OR (95% CI)	P value
Sex (F vs. M)	1.161 (1.005–1.341)	0.043	1.007 (0.860–1.178)	0.934
Age ≤ 2 vs others	0.165 (0.134–0.202)	<0.001	0.738 (0.564–0.965)	0.026
Age 3–5 vs others	0.464 (0.388–0.555)	<0.001	1.413 (1.155–1.728)	0.001
Age ≥ 6 vs others	4.626 (3.964–5.400)	<0.001	0.900 (0.756–1.071)	0.236
CTAS	0.415 (0.376–0.459)	<0.001	0.528 (0.470–0.592)	<0.001
Abdomen	1.702 (1.357–2.133)	<0.001	1.394 (1.136–1.711)	0.001
Back	2.951 (1.348–6.461)	0.007	1.320 (0.764–2.283)	0.320
Chest	2.750 (1.573–4.809)	<0.001	0.877 (0.557–1.382)	0.571
GU/Pelvic	0.733 (0.426–1.262)	0.263	1.147 (0.620–2.123)	0.662
Head and Neck	0.345 (0.297–0.400)	<0.001	1.119 (0.951–1.315)	0.176
Lower Ext	2.010 (1.634–2.473)	<0.001	0.673 (0.543–0.834)	<0.001
Upper Ext	1.506 (1.247–1.818)	<0.001	(0.900 (0.741–1.093)	0.288
Other	0.498 (0.193–1.289)	0.151	1.259 (0.413–3.837)	0.685

For the CTAS, we did not use a cut off. Rather, it is the OR for each 1 unit increase in CTAS score.

**Table 4:** Predictor characteristics of pediatric patients in multivariable model for pain assessment and treatment.

Predictor	Pain Assessed		Pain Treated	
	OR (95% CI)	P value	OR (95% CI)	P value
Sex (F vs. M)	1.012 (0.859–1.193)	0.882	0.957 (0.814–1.125)	0.596
**Age** ^**+**^				
Age ≤ 2	0.148 (0.117–0.188)	<0.001	0.782 (0.588–1.041)	0.092
Age 3–5	0.410 (0.333–0.505)	<0.001	1.413 (1.139–1.752)	0.002
**Acuity**				
CTAS	0.391 (0.351–0.436)	<0.001	0.528 (0.470–0.594)	<0.001
Location of pain*				
Back	1.691 (0.722–3.958)	0.226	1.059 (0.593–1.893)	0.846
Chest	1.528 (0.821–2.844)	0.181	0.685 (0.419–1.121)	0.132
GU/Pelvic	0.671 (0.347–1.295)	0.234	1.062 (0.552–2.046)	0.856
Head and Neck	0.615 (0.474–0.798)	<0.001	0.958 (0.756–1.214)	0.722
Lower Ext	1.547 (1.13–2.111)	0.006	0.677 (0.513–0.893)	0.006
Other	0.534 (0.171–1.671)	0.281	0.981 (0.306–3.149)	0.974
Upper Ext	1.157 (0.861–1.553	0.333	0.836 (0.644–1.085)	0.178

+ Reference category = children ≥ 6 years

*Reference for Location of pain = abdominal pain

## Discussion

Pain assessment is important and should be prioritized in pediatric patients in the ED. The updated International Federation of Emergency Medicine (IFEM) Standards of Care for Children in the ED states that pain in children should be assessed and treated within 30 min of arrival at the ED [15]. Italy has placed legal precedence on pediatric pain assessment, and the UK Royal College of Emergency Medicine has established guidelines and clinical standards for pediatric pain management in the ED [14, 16]. The Health Standards Organization of Canada has created the Pediatric Pain Management standard to help create organizational policies which inform clinicians of the best care practices [17]. Guidelines and policies are in place to help clinicians understand the importance of pain assessment and treatment but enabling them seems to be a challenge.

Our study was designed to determine the rates of pain assessment and treatment at triage and to identify the predictors of each outcome. Assessing pediatric pain is challenging considering the range of verbal, cognitive, and psychological development observed in children aged under 18 years. Furthermore, centers that do not commonly evaluate pediatric patients are uncomfortable with different pain scales or appropriate medications. A recent study in Quebec, Canada, showed that only 55% of children were screened for pain in the ED, thereby emphasizing the need to improve pediatric pain assessment [19]. Poor assessment results in a lack of treatment, and previous studies have shown that only 30%–40% of pediatric patients receive analgesia [2, 3, 21]. Our study had a higher rate of pain assessment (74.2%), but only 24.9% of those assessed received treatment. Pain is considered the fifth vital sign and is incorporated into the electronic CTAS (eCTAS) triaging process since 2017 at all five sites, thereby explaining the higher rate of pain assessment at triage. Pain treatment, however, has not changed.

To the best of our knowledge, this is the first study to examine the predictors of triage pain assessment and subsequent management from the perspective of a community ED. Previous studies have primarily been conducted at regional pediatric centers, which account for a minority of hospitals across Canada. Ferrante and colleagues looked at pain management across Italian EDs with 74% of their samples belonging to pediatric centers [3]. Results showed that only 26% of children were assessed for pain at triage. Similarly, Ganzijeva et al. looked at pain assessment and management at a tertiary care center before and after changes regarding pain management were implemented [5]. They showed that the rate of pain assessment increased from 84% to 94% when there was an intervention focusing on pain assessment. In our case, the assessment rate was 74.2%, which was significantly lower than their baseline. This phenomenon may be attributed to a higher vigilance on pain assessment to begin with and the pediatric specific site. Drendel et al. developed a prediction model for pain assessment in pediatric patients, and while they included multiple sites across the country it was unclear if any were pediatric centers. Their study found that only 44% of patients had a documented pain score, which was significantly lower than that described in the current and other studies.

Several assessment tools based on behavioral (FLACC, MAPS), pictorial (FACES), and numeric (visual analog scale) scales are available for pediatric pain assessment, and determining the best tool is challenging to identify for younger children [22]. Recognizing there are different pain scales based on the developmental spectrum of the child, we divided the age range into three categories. The first category included children aged 6 years and above. Evidence supports that children in this age group can quantify their pain either on a numerical or visual analogue scale [23–25]. The second group included infants and young toddlers (≤ 2 years). This age group is considered the most challenging when performing pain assessments as they are primarily assessed using behavioral characteristics. The final category included those between the ages of 3 and 5 years. At this age stage children can typically begin to articulate their experience of their pain with some notion of intensity. Not surprisingly, when compared with children ≥ 6 years, the other two categories were less likely to be assessed for pain. An unanticipated result was to find children aged 3–5 years were more likely to be treated for pain compared to those ≥ 6 years (OR. 1.413; 95% CI 1.139–1.752) in the prediction model.

The CTAS triage acuity categories patients from a scale of 1–5, with 1 being the highest acuity and 5 the lowest. Our study showed that patients with higher acuity were more likely to be assessed and treated. This is not surprising as most EDs prioritize trauma patients or those with significant injuries for immediate care, including appropriate assessment.

In our multivariable prediction model, we found that head and neck pain was less likely to be assessed compared with abdominal pain (OR 0.615; 95% CI: 0.474–0.798). Notably, many children are brought to the ED with head injuries, the majority of which are categorized as minor head injuries. The lack of symptoms (neurological or secondary to significant pain) could have biased the triage nurse to skip the pain assessment. In contrast, those with lower extremity pain were more likely to be assessed for pain (OR 1.547; 95% CI 1.13–2.111) and treated. Although upper extremity injuries are more common, they are also easier to immobilize; therefore, the children with arm pain would likely have been in a more comfortable position. While this may not explain the lack of assessment, it may have contributed to the lower rate of treatment.

This study aimed to evaluate the rates of pain assessment and management at triage and to identify the predictors of each outcome in community EDs. Results showed that with the implementation of eCTAS, the rate of pain assessment increased (74.2% in our case), but there was little difference in pain management. This study had some limitations. First, it is limited by the inherent bias of retrospective studies, which has been described in previous studies [26–28]. Although there were data on patients who received analgesia, there was no documentation on patients who refused such a treatment. Either the patient or their family member might have declined treatment with analgesia, and this could reflect the lower administration rate. Second, the pain severity was not examined, which might have affected the treatment. However, we observed that all types of minor pain could be easily treated with oral ibuprofen or acetaminophen. Nonpharmacological treatment, such as splinting for musculoskeletal injuries, was not evaluated. Again, a certain level of treatment would be expected in certain cases, such as splinting an injured limb and sending the patient for diagnostic imaging studies. Third, we examined all pediatric patients. Recognizing the challenges of assessing infants with limited verbal skills, it is not surprising that these children are vulnerable to inappropriate assessments. While we appreciate that there were assessments captured, we do not have information regarding whether the most age-appropriate pain scale was used for each child.

Despite the higher rate of pain assessment at triage, further improvement is required. With the implementation of pain as a modifier in eCTAS, > 95% of patients should undergo pain assessment. Furthermore, the prediction model clearly identified the challenge of assessing infants and toddlers in a community ED. Therefore, more education and training are required to help triage nurses become comfortable and confident when evaluating younger children. Finally, pain assessment was found to vary based on the location of pain; hence consistent means of assessment regardless of the where the pain is located need to be developed and promoted. If the assessment rates increase, then treatment strategies should follow. Targets should be set to manage pain in at least 80% of patients while aiming to provide treatment within 30 min of arrival [15].
